# Longitudinal Associations of Body Fatness and Physical Fitness with Cognitive Skills in Preschoolers

**DOI:** 10.3390/children11050526

**Published:** 2024-04-27

**Authors:** Kirkke Reisberg, Eva-Maria Riso, Liina Animägi, Jaak Jürimäe

**Affiliations:** 1 Department of Physiotherapy and Environmental Health, Tartu Health Care College, 50411 Tartu, Estonia; liinaanimagi@nooruse.ee; 2 Institute of Sport Sciences and Physiotherapy, Faculty of Medicine, University of Tartu, 51008 Tartu, Estonia; eva-maria.riso@ut.ee (E.-M.R.); jaak.jurimae@ut.ee (J.J.)

**Keywords:** body fatness, cardiorespiratory fitness, muscular fitness, cognitive skills, preschool children

## Abstract

A good cognitive status predicts academic, professional, and health outcomes. However, longitudinal data regarding the associations of body fatness, physical fitness, and cognition are relatively scarce and mixed. The purpose of this longitudinal study was to investigate whether body fatness, cardiorespiratory fitness (CRF), and muscular fitness (MF) in preschool are associated with cognitive skills in the first grade of school. A total of 133 South Estonian children whose age was 6–7 years were recruited from 13 kindergartens and again at 7–8 years after they had entered school. Body fat percentage (BF%), CRF, MF as the mean of z-scores of relative upper-limb strength, standing long jump results, and cognitive skills (verbal, conceptual, and perceptual) were studied. There were no associations between BF% and CRF in preschool with perceptual, conceptual, or verbal skills in school in boys and girls. In boys, a higher MF in preschool was associated with higher verbal skills (β = 0.293, *p* = 0.021) in school after adjustment for confounders. Cognitive skills at baseline seemed to be frequently associated with cognitive performance in school. In conclusion, higher MF in preschool was associated with better verbal skills in the first grade of school in boys but not in girls. Body fatness and cardiorespiratory fitness in preschool were not associated with cognitive skills in school.

## 1. Introduction

The prevalence of overweight and obesity among children and youth is on rise [1]. It raises concerns over the impact of overweight and obesity not only on children’s physical health [2,3] but potentially also on their cognitive performance; young individuals with obesity and metabolic syndrome show certain differences in brain structure (e.g., frequently a reduced gray matter volume) and functional connectivity compared to controls with healthy weight [4]. Additionally, children with obesity tend to have a lower self-esteem [5], while lower self-esteem in turn predicts poorer cognitive performance [6]. Accordingly, overweight and obesity among young people might affect their cognitive health.

There are studies to suggest inverse relationships between body fatness and cognitive skills in early childhood [7,8,9], while other investigations have not detected associations between body fatness and cognition [8,10,11]. For example, Haapala et al. [9] found a negative relationship between body fat percentage (BF%) and reading fluency and reading comprehension in 6–8-year-old boys. Martin et al. [8] observed that a higher body mass index (BMI) in 3-year-old boys was associated with worse visuospatial skills, yet not with expressive language skills or reasoning skills at 5 years. Flores et al. [11] reported that obesity was not associated with arithmetical performance among 3–6-year-old children. In addition, previous studies disagree on whether higher regional or whole-body fat content predicts worse cognitive performance [7,12,13]. Higher abdominal fat deposition was associated with worse relational memory but not with item memory in children with overweight or obesity at 7–9 years. Whole-body fat content was not associated with relational or item memory. Also, no associations between body fatness parameters and memory tasks existed in children with healthy weight [12]. Likewise, Raine et al. [13] found that especially in 8–9-year-old children with obesity, a reduction in visceral adipose tissue only over the course of 9 months was associated with increased inhibitory control [13]. However, higher whole-body and abdominal body fatness predicted inferior executive function, resistance to distraction, and gestalt processing among 7–11-year-old children with overweight [7]. Therefore, very few observational studies have verified the gender-specificity of these associations, finding a negative association between body fatness and cognition among preschool-aged [8] and first-grade boys [9] but not in girls. It has been found that a decrease in obesity status had positive associations with some cognitive skills in preschool-aged girls [8]. Regarding these mixed results and the paucity of longitudinal studies [8,10], the current study aims to explore the possible longitudinal associations between body fatness in preschool-aged boys and girls separately with their cognitive skills in school.

Worldwide, there is evidence for a constant decline in physical activity from early childhood into adolescence [14,15,16] in parallel with an increasingly sedentary lifestyle [15,17], which may have a possible impact on children’s physical fitness levels since PF is regarded as an indirect reflection of long-term physical activity [18]. A study has demonstrated a larger hippocampus among children who had higher cardiorespiratory fitness (CRF); they also performed better in a hippocampal-dependent relational memory task, whereas associations between CRF, nucleus accumbens volume, and memory were missing. Also, no association between CRF and whole-brain size was found [19]. Ortega et al. [20] performed a shape analysis of subcortical brain nuclei among children and found that CRF was mostly associated with enlargements, upper-limb strength was mainly associated with contractions, and only a few positive or negative associations between lower-limb strength and the shape of subcortical brain nuclei were present.

Similarly, there is a lack of uniformity in findings on the associations between PF and cognition in children and adolescents; CRF and muscular fitness (MF) seem to be associated with some cognitive skills, but not with others. For example, in 4–6-year-old children, CRF at baseline was related to improvements in attention and not to working memory during a 9-month period [21]. Also, 9-year-old children with higher CRF had better visual discrimination [22] but not better reaction time compared to children with lower CRF [23]. Moreover, CRF at 6–9 years did not predict non-verbal cognitive performance two years later in boys or girls [10]. Regarding MF, neither standing long jump (SLJ) nor handgrip strength at 7–9 years predicted non-verbal cognitive performance further in childhood and adolescence [24]. Meanwhile, Syväoja et al. [25] reported that higher MF was indirectly associated with higher math outcomes through visuospatial working memory only among adolescent girls [25]. In addition to the contradictory findings described above, the number of studies on the associations between CRF, SLJ, and handgrip strength, either addressed separately or as a compound MF score, in children is relatively small, which explains the lack of longitudinal studies in preschoolers [21,26] or primary schoolers [10,24,27]. Accordingly, it is important to study the associations of CRF and MF in preschool-aged boys and girls with their cognitive skills in school.

Our work intends to extend the understanding of the relationships between the body fatness and physical fitness of preschoolers with their verbal, conceptual, and perceptual skills in school, as cognitive skills may have significant educational, occupational and health outcomes [28]. The specific aim of the present study was to investigate whether body fatness, cardiorespiratory fitness, and muscular fitness in preschool are associated with cognitive skills in the first grade of school in boys and girls.

## 2. Materials and Methods

### 2.1. Participants

This longitudinal two-phased study was conducted in Tartu County in South Estonia. Altogether, 400 children aged 6–7 years from thirteen kindergartens, and their parents, were invited to participate in 2016 in the first wave and 284 families accepted the invitation. Families were again asked to participate in 2017 (second wave), when the children were in grade one in school. Two hundred families gave their consent and assent to participate. Complete data for statistical analysis were available for 133 children (67 boys, 66 girls). Additionally, each parent completed a short questionnaire reporting their educational attainment (categorized as basic, general secondary/vocational, or higher). More highly educated parents’ data were taken into account. The Research Ethics Committee of the University of Tartu (reference 254/T-13 and 266/T-8) approved this study. This study is in agreement with the ethical principles of the Declaration of Helsinki.

### 2.2. Assessment of Body Composition

Body height was measured using a portable stadiometer (Seca 213, Hamburg, Germany) to the closest 0.1 cm. Body weight was measured using a digital medical scale (A&D Instruments, Abington, UK) to the nearest 0.05 kg. BMI was calculated as body weight (kg)/body height (m)^2^. Children with overweight and obesity were identified by age- and gender-specific BMI cutoff values [29]. Skinfold thicknesses (triceps and subscapular) were measured to the nearest 0.2 mm in triplicate with a Holtain caliper (Crymych, UK) according to a standardized protocol [30,31]. Slaughter et al.’s [32] equations for children and adolescents aged 6–17 years were used to calculate BF%.

### 2.3. Assessment of Physical Fitness

To measure CRF, a 20 m shuttle run test was applied [33] based on a standardized protocol [34]. In brief, each child was instructed to run between two marked lines over a 20 m course in time with taped audio signals. The test started at 8.5 km/h, increased by 0.5 km/h after every minute. The test ended when the child could not keep up with the pace anymore. The number of completed laps was taken into account. The test was performed once.

To determine upper-limb muscular fitness (MF), a handgrip strength test was conducted using digital dynamometer (Digital TKK 5401, Grip D, Takei, Tokyo, Japan). The test was conducted in a standing position, shoulder slightly abducted and elbow extended. The child squeezed the dynamometer for 2–3 s. The best value of two attempts with each hand was taken into account. Handgrip strength was calculated in kg as the average for the right and left hand [35]. Relative upper-limb MF kg/body mass (in kg/kg) was used [36].

To determine lower-limb MF, the SLJ test (cm) was used. The child stood on a line, with the feet slightly apart. They performed two-footed takeoffs, landing as far as possible [37]. The best of two trials was taken into account [38].

A muscular fitness (MF) z-score was computed from the combination of gender-specific standardized measures of relative upper-limb MF (kg/kg) and lower-limb MF (cm). The MF z-score was computed as the mean of the two z-scores (relative upper-limb MF and lower-limb MF), where z-score = (value-mean)/standard deviation [36].

### 2.4. Assessment of Cognitive Skills

A Modified Boehm Test of Basic Concepts—Third Edition (Boehm-3) was applied to measure cognitive skills [39]. This test has been adjusted and validated for use in Estonia [40,41]. A progressive matrix test was applied to test the children’s perception; accordingly, each child had to select the right picture to provide a structured sequence of pictures. In order to assess conceptual and verbal skills, the children had to identify a picture that matched the sentence read by examiner. The children’s conceptual comprehension of adverbs, as well of where objects were located, were assessed to measure conceptual skills. Verbal skills were assessed as the child’s comprehension of the Estonian language.

### 2.5. Statistical Analysis

The analyses were run separately for boys and girls. Continuous variables were examined for normality using the Shapiro–Wilk test. We compared basic characteristics using the Student *t*-test for normally distributed continuous variables and applied the Mann–Whitney U test for other continuous variables, while for categorical variables (prevalence of overweight and obese, parental education), the chi-square test was used. Associations between BF%, CRF, and MF z-score in preschool with verbal, conceptual, and perceptual skills in school were examined using multiple linear regression analysis. Baseline values of exposures (BF%, CRF, MF z-score), baseline outcomes (verbal, conceptual, and perceptual skills), age, and parental education were included in the adjusted model. Adjustments were made for well-documented confounding variables such as age, parental education, and baseline cognitive skills [10,21,42,43]. Multicollinearity was not a problem, since the variance inflation factors between the variables were less than 5. A significance level of *p* < 0.05 was regarded as statistically significant. SPSS software (version 20.0; SPSS, Inc., Chicago, IL, USA) was used.

## 3. Results

### 3.1. Baseline Characteristics of Children

Boys were taller (*p* < 0.001), heavier (*p* = 0.010), and had greater handgrip strength (*p* = 0.007) than girls. Boys also had a lower BF% than girls (*p* < 0.001) (Table 1).

### 3.2. Associations of BF% in Preschool with Cognitive Skills in School

In boys, BF% in preschool was not associated with cognitive skills in school. In the adjusted model, verbal (R^2^ = 0.217, *p* = 0.002) and perceptual (R^2^ = 0.300, *p* < 0.001) skills in preschool were positively associated with verbal and perceptual skills (both *p* < 0.001) in school, respectively (Table 2).

In girls, BF% in preschool was negatively associated with perceptual skills (R^2^ = 0.070, *p* = 0.036) in school. In the adjusted model, conceptual (R^2^ = 0.195, *p* = 0.004) and verbal (R^2^ = 0.206, *p* = 0.003) skills in preschool were positively associated with conceptual (*p* = 0.002) and verbal (*p* = 0.001) skills in school, respectively. Additionally, age in preschool was negatively associated with verbal skills (*p* = 0.024) in school. Also, higher parental educational attainment in preschool (R^2^ = 0.292, *p* < 0.001) was associated with higher perceptual skills (*p* < 0.001) in school (Table 2).

### 3.3. Associations of CRF in Preschool with Cognitive Skills in School

In boys, CRF in preschool was positively associated with perceptual skills (R^2^ = 0.081, *p* = 0.033) in school. In the adjusted model, verbal (R^2^ = 0.151, *p* = 0.018) and perceptual (R^2^ = 0.259, *p* < 0.001) skills in preschool were positively associated with verbal (*p* = 0.003) and perceptual skills (*p* < 0.001) in school, respectively (Table 2).

In girls, CRF in preschool was positively associated with conceptual skills (R^2^ = 0.073, *p* = 0.039) in school. In the adjusted model, conceptual (R^2^ = 0.220, *p* = 0.004) and verbal (R^2^ = 0.166, *p* = 0.014) skills and parental education (R^2^ = 0.278, *p* = 0.001) in preschool were positively associated with conceptual (*p* < 0.001), verbal (*p* = 0.013), and perceptual skills (*p* < 0.001) in school, respectively. In addition, older age in preschool was associated with lower verbal (*p* = 0.023) and perceptual (*p* = 0.022) skills in school (Table 2).

### 3.4. Associations of Muscular Fitness in Preschool with Cognitive Skills in School

In boys, MF in preschool was positively associated with verbal skills (R^2^ = 0.074, *p* = 0.024) in school. In the adjusted model, the association between MF in preschool and verbal skills in school remained positive (*p* = 0.021). Additionally, conceptual (R^2^ = 0.095, *p* = 0.066), verbal (R^2^ = 0.236, *p* = 0.002), and perceptual (R^2^ = 0.248, *p* = 0.001) skills in preschool were positively associated with conceptual (*p* = 0.048), verbal (*p* = 0.004), and perceptual skills (*p* < 0.001) in school, respectively (Table 2).

In girls, MF in preschool was not associated with cognitive skills in school. In the adjusted model, higher conceptual (R^2^ = 0.208, *p* = 0.005) and verbal (R^2^ = 0.214, *p* = 0.004) skills and parental education (R^2^ = 0.298, *p* < 0.001) in preschool were associated with higher conceptual (*p* = 0.001), verbal (*p* = 0.008), and perceptual skills (*p* < 0.001), respectively. In addition, older age in preschool was associated with lower perceptual (*p* = 0.009) skills in school (Table 2).

## 4. Discussion

The transition from preschool to school is a crucial turning point in children’s lives. School readiness typically sets standards for several developmental domains, including children’s nutritional status, overall PF level, and motor and cognitive development [44,45]. The preschool period should prepare children for successful adaptation to school entry in order to fulfil school requirements [44]. Therefore, this study aimed to investigate whether body fatness, cardiorespiratory fitness, and muscular fitness in preschool are associated with cognitive skills in the first grade of school. The main findings were as follows: (1) BF% in preschool was not associated with conceptual, verbal, and perceptual skills in the first grade of school in boys and girls after adjustment for potential confounding factors; (2) CRF in preschool was not associated with conceptual, verbal, and perceptual skills in school in boys and girls when adjusted for possible confounding factors; (3) higher MF in preschool was associated with higher verbal skills in school only in boys after adjustment for potential confounding factors. It was also observed that cognitive skills at preschool were frequently positively associated with cognitive performance at follow-up in the first grade of school both in boys and girls, although there were some gender differences. Additionally, higher parental educational attainment at baseline was associated with higher perceptual skills at follow-up in girls. Correspondingly, Riso et al. [43] concluded that preschoolers from more highly educated families had higher conceptual and verbal skills. Interestingly, younger age in preschool was occasionally associated with higher verbal or perceptual skills in school only among girls.

In the unadjusted analysis, we found that skinfold thickness-derived BF% in preschool was negatively associated with perceptual skills in school among girls. However, once adjusted for confounding factors, such as age, parental education, and cognitive skills at baseline, no associations between BF% in preschool and conceptual, verbal, or perceptual skills in the first grade of school existed in boys or girls. Prior studies in children show rather mixed results in terms of correlations between BF% or obesity and cognitive measures [7,9,10,13,46]. Haapala et al. [9] reported a weak inverse association between dual-energy x-ray absorptiometry (DEXA)-measured BF% and the ability to read with proper speed and accuracy, as well as the ability to understand the text that was read, among 6–8-year-old boys after controlling for age and parents’ education. Variation in motor performance between boys with lower and higher BF% explained these gender-specific associations to a high degree [9]. In a longitudinal study, in 3-year-old boys, obesity predicted inferior pattern recognition, but not naming vocabulary or reasoning skills at 5 years after controlling for several confounding factors. “Growing out” of obesity between the ages of 3 and 5 years was positively correlated with reasoning skills in 5-year-old girls [8]. In children at 8–9 years of age, only the extent of reduction in visceral fat mass over the course of 9 months was associated with increased inhibitory control. These associations were particularly obvious among obese children [13]. However, Davis and Cooper [7] showed that both whole-body and abdominal body fatness were negatively associated with executive function, resistance to distraction, and gestalt processing in children with overweight aged 7–11 years after adjustment for gender, race, and parental educational attainment [7]. Consistent with the present study, Haapala et al. [10] demonstrated that bioelectrical impedance method-detected BF% at 6–9 years did not predict non-verbal reasoning skills after two years in either gender after controlling for cognitive skills at baseline. Meanwhile, one study even suggested that not overweight but underweight at an early age predicted worse cognitive and academic performance once adjusted for early behavior, cognitive skills, and socioeconomic status [46]. The variability in the results of these studies might come from differences in the weight status of the studied children, different methods applied to investigate body composition, different cognitive tasks involved in the studies, sociodemographic differences across countries, and differences in adjustments for confounders. In current study, body fatness in preschool was not independently associated with cognitive skills in grade one in boys or girls.

Studies by Chaddock et al. [19] and Ortega et al. [20] in preadolescent children demonstrate that the associations between PF items and brain structures might be specific, where certain brain structures seem to react to a certain PF item either by enlargement or contraction, the exact impact of which on the functions of the brain, including on cognitive performance, needs to be further explored [20]. Accordingly, there seems to be a variability in associations between PF and cognition in children and adolescents across studies.

In our study, higher CRF in preschool was associated with higher conceptual skills in girls and higher perceptual skills in boys a year later. However, these associations disappeared after controlling for confounding variables like age, parental education, and cognitive skill at baseline. More laps in a 20 m shuttle run test in preschool were related to better perceptual skills in school. However, this association disappeared after adjusting for gender and age; additionally adjusting for children’s sports club attendance and parental education did not change this association [26]. In general, there seems to be a lack of consistency in the studies exploring the associations between CRF and cognition at a young age. A cross-sectional study showed that CRF was linked to better working memory performance in boys but not in girls aged 8–11 years [47]. Children at 9 years of age with high CRF levels estimated using a 20 m shuttle run test displayed better outcomes in a visual discrimination task [22] and demonstrated higher accuracy in inhibitory control tasks than children who had lower CFR levels, although reaction times were not different between the groups [23]. Longitudinal research has also demonstrated mixed findings on the relationships between CRF and cognitive skills. Specifically, Chaddock et al. [27] showed that children with higher peak oxygen consumption at 9–10 years had superior response accuracy in compatibility conditions, yet not in incompatibility conditions on a flanker test at baseline and follow-up testing one year later, compared with less fit children. In addition, a shorter compatible and incompatible reaction time for children with higher levels of fitness was observed [27]. Niederer et al. [21] reported that baseline 20 m shuttle run test results were associated with improvements in attention but not with working memory during a 9-month period among children aged 4–6 years after controlling for several confounding factors [21]. To explore the association between CRF and cognition, Haapala et al. [10] applied a Raven’s progressive matrices test (RPM) test, which has methodological similarities to the non-verbal perceptual reasoning skills testing applied in our study. Comparably to our results, cycle ergometer-assessed exercise capacity at 6–9 years was not associated with RPM scores after two years, nor with changes in RPM scores during a two-year period in boys or girls after controlling for baseline RPM score, age, and study group. In accordance with the current study, baseline cognition strongly predicted cognitive functioning at two-year follow-up among both genders [10].

Differences in CRF and cognition testing methodology and the confounding variables included may modify the associations between CRF and cognitive performance, and hence clear up the diversity observed in the results of the studies. However, longitudinal research on the relationships between fitness items other than CRF and cognitive functioning is limited. The current study demonstrated that higher MF in preschool was associated with higher verbal skills in school independent of confounders in boys, with no such association observed for females. Syväoja et al. [25] detected that higher MF, calculated as the sum of upper-limb and abdominal strength, was indirectly associated with higher outcomes in mathematics through visuospatial working memory in 12–17-year-old girls but not in boys. Additionally, an indirect path from compound fitness z-score (including the six-minute-run, SLJ, and the jumping sideways task) via executive functions among 5–7-years preschoolers to mathematical and reading achievement 1.5 years later in school has been reported [48]. Higher SLJ relative to fat-free mass in preschool, but not relative handgrip strength parameters, were associated with superior perceptual skills in school [26]. After controlling for age, gender, pubescence, intervention group, menstrual age, mother’s age, family earnings, and parity, Lima et al. [24] found that at 7–9 years, out of all the PF components tested in children, only speed and agility, and manual dexterity were related to later cognitive skills on RPM at middle childhood and adolescence, respectively. However, 50 m dash and outcomes in a box and block test were not related to cognition after additionally controlling for prenatal, neonatal, and child fitness measures. Baseline SLJ or handgrip strength or sit-ups were not associated with later cognitive performance.

This study has some limitations. Compared with prior studies, our relatively smaller sample size may also explain the non-significant results reported in the current study. However, the number of participants in our study was comparable to corresponding research in this field [9,11,47,48]. Body fat content was measured indirectly by measuring skinfold thickness, and although Slaughter’s equation has fairly high validity with DEXA for calculating BF% in children, DEXA is still considered the gold standard in body composition assessment [49]. Although we adjusted our statistical analysis for some confounding factors, we cannot exclude the possibility of residual confounders due to genetic, socioeconomic, or nutritional factors. On the other hand, the strengths of our study are its longitudinal design and its use of standardized PF and cognitive skills testing.

## 5. Conclusions

We extended previous research by demonstrating that in our model, in which several independent variables were simultaneously included, cognitive skills in preschool seemed to be frequently associated with cognitive skills in the first grade of school, and only a higher MF compound score in preschool was independently associated with higher verbal skills in school among boys.

## Figures and Tables

**Table 1:** Basic characteristics.

Variable	Boys	Girls	*p*
Age (years) ^1^	7 (1)	6 (1)	0.240
Height (cm)	127 (5.4)	124 (5.7)	**<0.001**
Weight (kg) ^1^	26 (6.5)	23.5 (5.6)	**0.010**
BMI (kg/m^2^) ^1^	16.1 (2.5)	15.4 (2)	0.323
Prevalence of overweight and obese (%)	17.9	9.9	0.170
BF%	19.8 (6.3)	21.4 (5.9)	**<0.001**
**Parental education (%)**			0.765
Lower secondary	1.4	3	
Post-secondary or vocational	15.7	17.9	
University degree	82.9	79.1	
**Physical fitness tests**			
20 m shuttle run (laps) ^1^	18 (19.3)	18.5 (8.5)	0.383
Handgrip strength (kg) ^1^	11.5 (3)	9.9 (2.6)	**0.007**
Handgrip strength/weight (kg/kg)	0.4 (0.1)	0.4 (0.1)	0.299
Standing long jump (cm)	125 (18)	119 (18)	0.061
Muscular fitness z-score	0.01 (0.5)	−0.01 (0.5)	0.797
**Modified Boehm-3 test**			
Perceptual skills (max score 10) ^1^	8 (3)	7 (5)	0.201
Conceptual skills (max score 17) ^1^	14.5 (2)	14 (2.8)	0.418
Verbal skills (max score 9) ^1^	5.5 (1)	6 (1)	0.650

Data are from Student’s *t*-test or the Mann–Whitney U-test, or the chi-square test for categorical variables, and are presented as means (standard deviations), medians (interquartile ranges ^1^), or percentages (%). BMI—body mass index, BF%—body fat percentage. Figures marked in bold indicate statistically significant differences.

**Table 2:** Multiple regression analysis demonstrating the associations of body fatness and physical fitness in preschool with cognitive skills in school.

	Cognitive Skills in School											
	Conceptual Skills				Verbal Skills				Perceptual Skills			
	Boys (*n* = 66)		Girls (*n* = 67)		Boys (*n* = 66)		Girls (*n* = 66)		Boys (*n* = 66)		Girls (*n* = 67)	
Variables in Preschool	β	*p*	β	*p*	β	*p*	β	*p*	β	*p*	β	*p*
Unadjusted												
BF%	−0.075	0.599	0.221	0.082	−0.155	0.227	−0.016	0.901	−0.079	0.539	−0.265	**0.036**
Adjusted												
BF%	0.033	0.801	0.190	0.130	−0.082	0.500	−0.040	0.746	−0.172	0.139	−0.184	0.119
Age	0.168	0.199	−0.092	0.449	0.064	0.592	−0.284	**0.024**	−0.151	0.186	−0.203	0.081
Cognitive skills *	0.177	0.195	0.396	**0.002**	0.467	**<0.001**	0.416	**0.001**	0.574	**<0.001**	0.189	0.103
Education	0.254	0.073	0.149	0.238	0.113	0.358	0.069	0.579	−0.166	0.153	0.493	**<0.001**
Unadjusted												
CRF	−0.070	0.607	0.270	**0.039**	0.033	0.807	0.164	0.213	0.285	**0.033**	0.067	0.617
Adjusted												
CRF	−0.123	0.367	0.065	0.637	0.034	0.791	−0.039	0.786	0.227	0.059	−0.052	0.696
Age	0.182	0.190	−0.131	0.307	0.091	0.500	−0.306	**0.023**	−0.129	0.290	−0.288	**0.022**
Cognitive skills *	0.291	0.061	0.450	**<0.001**	0.419	**0.003**	0.341	**0.013**	0.535	**<0.001**	0.201	0.109
Education	0.037	0.805	0.140	0.329	0.073	0.574	0.084	0.564	−0.085	0.471	0.510	**<0.001**
Unadjusted												
Muscular fitness	−0.150	0.270	0.056	0.674	0.302	**0.024**	−0.207	0.116	0.182	0.180	0.152	0.252
Adjusted												
Muscular fitness	−0.142	0.289	0.021	0.875	0.293	**0.021**	−0.223	0.102	0.097	0.432	0.255	0.051
Age	0.155	0.255	−0.110	0.419	0.127	0.319	−0.229	0.095	−0.090	0.467	−0.347	**0.009**
Cognitive skills *	0.303	**0.048**	0.460	**0.001**	0.380	**0.004**	0.349	**0.008**	0.531	**<0.001**	0.219	0.072
Education	0.042	0.776	0.140	0.294	0.081	0.515	0.084	0.512	−0.073	0.551	0.448	**<0.001**

* Baseline cognitive skill (conceptual, verbal, or perceptual skill) score was entered in the model with respective cognitive skill at follow-up. β—standardized regression coefficient; BF%—body fat percentage; CRF—cardiorespiratory fitness. Figures marked in bold indicate statistically significant associations.

## Data Availability

The datasets used in this study are available from the corresponding author upon reasonable re-quest due to privacy and ethical reasons.
